# Insight on immune cells in rejection and infection postlung transplant

**DOI:** 10.1002/iid3.868

**Published:** 2023-07-12

**Authors:** Mingfeng Liao, Chaoxi Wang, Mingxia Zhang, Kun Qiao

**Affiliations:** ^1^ Guangdong Key Lab for Diagnosis & Treatment of Emerging Infectious Diseases Shenzhen Third People's Hospital Shenzhen Guangdong Province People's Republic of China; ^2^ Department of Thoracic Surgery Shenzhen Third People's Hospital Shenzhen Guangdong Province People's Republic of China

**Keywords:** immune cells, lung transplantation, obliterative bronchiolitis, rejection, tolerance

## Abstract

**Objective:**

The aim of this study is to provide a concise overview of the role of immune cells in rejection and infection after lung transplantation.

**Methods:**

Based on previous clinical and basic studies, the role of various types of immune cells in the development of rejection and infection after lung transplantation is summarized.

**Results:**

Immune cell functional status is strongly associated with common complications after lung transplantation, such as primary graft dysfunction, infection and occlusive bronchitis syndrome. Targeted balancing of immune cell tolerance and rejection is an important tool for successful lung transplantation.

**Conclusion:**

A comprehensive understanding of immune cell function and the mechanisms that balance immune tolerance and immune rejection may be a crucial factor in improving survival after lung transplantation.

## Graphical abstract

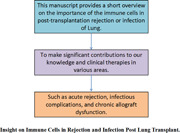

## 1 INTRODUCTION

Lung transplantation is a primary treatment for end‐stage lung failure resulting from various chronic lung diseases. Similar to other organ transplants, preventing rejection is the primary focus of treatment after lung transplantation. In allogeneic transplantation, two fundamental types of graft rejection can occur: Host‐versus‐Graft Reaction (HVGR) and Graft‐versus‐Host Reaction. HVGR is the most frequent type of reaction after lung transplantation, and its incidence is higher than in recipients of other solid organ transplants. Due to the lung being an open organ, the incidence of posttransplant infection is also relatively high.^1^

The treatment approach for posttransplant infection or rejection is entirely different, making it challenging to determine the appropriate dosage of immunosuppressive drugs for different patients. Excessive immunosuppressive therapy can lead to immunodeficiency, significantly increasing the incidence of infection and tumors. On the other hand, insufficient immunosuppressive therapy can lead to graft rejection, which is equally problematic.^2^

Recent years have seen improvements in the short‐term survival of lung transplant recipients, with the 3‐month survival rate reaching 90% and the 1‐year survival rate increasing to 81%. However, long‐term survival rates have remained virtually unchanged, with a median survival of 5.6 years. The 5‐year survival rate for lung transplant recipients is only about 50%, which is lower than that of other solid organ recipients.^3^ The main factors contributing to poor long‐term survival after lung transplantation are infection and/or chronic rejection of the lung graft.^4^ Immune monitoring and treatment after lung transplantation differs from other organ transplants, such as the liver and heart. Maintaining immune balance in lung transplant recipients using only immunosuppressive drugs is difficult.

In recent years, an emerging strategy involving targeting immune cells to induce immune tolerance has offered new possibilities for researchers and clinicians. This approach may lead to the development of more targeted and effective therapies that reduce the use of immunosuppressive drugs and associated complications such as infections and tumors. Which immune‐monitoring indicators are sensitive to rejection or infection? How can immune cells be used to induce immune tolerance and maintain a balance between rejection and infection? How do immune cells regulate immune rejection and induce immune tolerance after transplantation? These are important questions that require further exploration to improve the outcomes for lung transplant recipients.

To address these questions, we conducted a comprehensive search of the Science Citation Index Expanded over the last decade. Using the search formula ALL = (immune cell AND (lung transplantation) OR (lung rejection) OR (lung transplant rejection) OR (lung allograft tolerance) OR (lung graft rejection)), we identified 2431 relevant research papers, reviews, reports, and standards. We then carefully screened and summarized this literature to guide further studies on lung transplantation immunity. Our overview of the screened literature highlights the many advances that have been made in our understanding of immune cells and the challenges that remain.

## 2 NATURAL KILLER (NK) CELLS

NK cells are a crucial component of the innate immune system and play a critical role in the early response to infections and cancer. However, recent studies have revealed that NK cells also possess regulatory functions in adaptive immunity. One landmark study by Ruggeri et al.^5^ in 2002 demonstrated that NK cells can effectively suppress human hematopoietic graft versus host disease (GVHD) through interactions between the killer cell Ig‐like receptor (KIR) and its ligands. This finding established a significant link between NK cells and transplantation immunity, highlighting their potential as therapeutic targets for immune modulation in the context of lung transplantation.

Emerging evidence has demonstrated that NK cells can exert both pro‐ and anti‐inflammatory effects in the context of lung transplantation.

On the one hand, recipient (donor) NK cells can recognize the donor (receptor) cells via KIR, providing an auxiliary signal for T cell responses and thereby promoting immune rejection. NK cells can also recognize specific antibodies via Fc receptors and subsequently participate in immune rejection.^6^, ^7^ Additionally, cytokines and effector molecules secreted by NK cells, such as interferon γ (IFN‐γ) and tumor necrosis factor α (TNF‐α), can induce delayed‐type hypersensitivity, activate naive T cells, and promote the cell‐killing effect of cytotoxic T cells in the graft. Moreover, NK cells from recipients can express important surface molecules that induce donor cell death, such as granzyme and the death receptor FasL. Several studies have suggested that NK cells play a crucial role in alloimmune rejection, as evidenced by their increased presence in MHC‐incompatible lung transplantation models.^8^, ^9^

On the other hand, recipient NK cells can also contribute to immune tolerance in several ways. First, NK cells can promote allogeneic tolerance by eliminating donor‐derived antigen‐presenting cells (APCs). This occurs through the secretion of TNF‐α and perforin, which target donor‐derived dendritic cells (DCs) and promote their maturation and killing.^8^, ^9^ This inhibits the direct activation of APCs targeting T cells, leading to the induction of recipient immune tolerance.

Second, recipient NK cells can also suppress the reactive proliferation of CD8^+^ T cells, which are involved in transplant rejection. Studies in mouse lung transplantation models have shown that successful depletion of NK cells after induction of immune tolerance stabilizes the function of the graft, indicating a complementary role for NK cells in T cell‐mediated immune tolerance.^10^ To test the hypothesis that NK cells play a critical role in immune tolerance, Jungraithmayr conducted experiments using IL‐15Ra−/− mice to establish a model of lung transplant rejection. The results revealed that NK cells appeared in the graft earlier than T lymphocytes, suggesting that NK cells are involved in the early stages of transplant rejection. Furthermore, treatment of the recipient with IL‐15/IL‐15Ra complex was found to reduce T lymphocyte infiltration in the graft and improve lung graft function, providing further evidence of the role of NK cells in suppressing T‐cell response in allogeneic lung transplantation.^8^

Moreover, recent research has uncovered a fascinating discovery that a subset of mouse and human NK cells possess memory‐like properties similar to memory T cells. These memory NK cells can induce memory responses by responding to cytokines such as IL‐12, IL‐15, and IL‐18. The existence of memory NK cells was first documented by Cudkowicz et al. in 1964, who found that NK cells exhibited immunological memory and induced immune tolerance in a murine bone marrow transplantation model.^11^ Further studies have confirmed the existence of a subset of memory NK cells; however, it remains unclear whether these cells are antigen‐specific. Nonetheless, the collective evidence suggests that NK cells may play a crucial role in establishing immune tolerance. These findings provide new insights into the potential therapeutic applications of NK cells in preventing transplant rejection and other immune‐related diseases. Collectively, these findings suggest that NK cells may play a crucial role in establishing immune tolerance, providing insights into potential therapeutic targets for the prevention of transplant rejection.

It has been suggested that NK cells may be involved in posttransplant inflammation.^12^ Studies have identified the ratio of NKG2C^+^ NK cells in bronchoalveolar lavage (BAL) as a potential biomarker for assessing the risk of cytomegalovirus (CMV) viremia and quantifying the likelihood of CMV‐related graft injury leading to chronic lung allograft dysfunction (CLAD) or death. Remarkably, the ratio of NKG2C^+^ NK cells was found to increase before the first detection of viremia, with patients who had high levels of viremia (>1000 copies/mL) having almost three times higher ratios than those with no detectable viremia. These findings suggest that NK cells, particularly those expressing NKG2C, may be associated with posttransplant inflammation and provide new insights into the role of NK cells in lung transplant rejection.

NK cells play a critical role in the immune response following lung transplantation. They are involved in the recognition and elimination of donor‐derived cells and can modulate the activity of other immune cells such as T cells. However, the exact mechanisms underlying NK cell‐mediated rejection or tolerance in lung transplantation are not fully understood and require further investigation.

## 3 DCs

Donor‐derived plasmacytoid dendritic cells (pDCs) have been found to play a crucial role in graft rejection following lung transplantation. Researchers from the Netherlands, led by Paantjens, conducted a monthly analysis of leukocyte chimerism in 11 lung transplant recipients, focusing on B cells, monocytes, NK cells, and DC cells. They compared the proportion of chimeric cells in the recipients with the circulating cell composition of the donor. The results showed that while the proportion of myeloid dendritic cells (mDC1s and mDC2s) was higher in the recipients, the proportion of pDCs was significantly lower. Specifically, the proportion of pDCs in the peripheral circulation of the recipients was <1%, whereas in the donor it was 25%.^13^

Interestingly, the pDCs in the recipients expressed the chemokine receptor CCR9 and migrated toward the CCR9 ligand CCL25, which is involved in the homing of T cells and DC cells. In peripheral and mesenteric lymph nodes, CCR9^+^ pDCs were found to induce simple allogeneic CD4^+^ CD25^+^ T cells to differentiate into CD4^+^ CD25^+^ Foxp3^+^ regulatory T cells (Tregs). These Tregs could then inhibit antigen‐specific immune responses both in vitro and in vivo, as well as allogeneic CD4^+^ donor T cell‐induced acute graft‐versus‐host disease.^6^, ^14^

Recent studies have highlighted the negative regulation of regulatory T cells (Tregs) via the expression of the enzyme indoleamine 2,3‐dioxygenase by DCs.^15^ However, the specific mechanisms underlying this regulation are still not fully understood.

Furthermore, DC cells have been found to engage in bidirectional regulation with NK cells, as well as with T lymphocytes, which plays a critical role in graft rejection. By actively interacting with other immune cells, DC cells can influence the immune response to transplanted tissues and organs. The intricate interplay between DC cells and other immune cells underscores the complex nature of the immune response to transplants.

## 4 LYMPHOCYTES

Lymphocytes are vital in the rejection of lung transplants, with B lymphocytes being responsible for producing antibodies such as human leukocyte antigen (HLA) antibodies and donor‐specific antibodies (DSA), which are key contributors to acute and chronic rejection. Additionally, cytotoxic rejection is supported or carried out by helper T lymphocytes (Th) and CTL. The interplay between T and B lymphocytes is crucial in determining the direction of immune rejection.

### 4.1 B lymphocytes

In the past, it was believed that rejection of transplanted organs primarily occurred through cell‐mediated immunity. However, studies have revealed that B lymphocytes also play a crucial role in transplant rejection.^16^ In fact, their versatility in this process has become increasingly apparent over the last two decades.

In 2011, researchers observed changes in IgM and IgG levels in transplanted lungs within 14 days of the procedure, and found that the immune activity of IgM could predict acute rejection.^17^ Another study in 2012 reported a case of lymphocytic syndrome in a lung transplant patient, highlighting the risk of passenger lymphocyte syndrome in heart and lung transplant patients.^18^ This syndrome occurs when residual B lymphocytes in the donor tissue produce antibodies that destroy the recipient's red blood cells, leading to hemolysis and acute rejection. In 2013, Chad's group in the United States proposed that antibody‐mediated rejection (AMR) induces an acute onset of lung transplant rejection (the median time of 258 days after lung transplantation).^19^

Moreover, antibody‐mediated rejection (AMR) has been found to cause acute onset of lung transplant rejection, with a high risk of CLAD. Clinical, immunological and pathological features of AMR include acute graft failure and chronic obliterative bronchiolitis (OB), donor‐specific human leukocyte antigen antibodies (DSA) in the circulation and lung tissue, intermediate complement C4d deposition and inflammatory infiltration.^20^, ^21^, ^22^, ^23^ These findings highlight the close relationship between B lymphocytes and transplant rejection.

While B lymphocytes have traditionally been associated with transplant rejection, recent studies have highlighted the potential of regulatory B cells (Bregs) in promoting immune tolerance. Regulatory B cells (Bregs) are a unique subset of B cells that have been shown to promote the maturation of regulatory T cells (Tregs) and mediate immune tolerance through specific mechanisms. One of the central factors in this regulatory mechanism is believed to be the inhibitory cytokine interleukin‐10 (IL‐10), which is secreted by Bregs. In a study published in Transplantation in 2015, IL‐10‐producing B lymphocytes were found to be necessary for blocking the CD40/CD40L co‐stimulatory signal and establishing immune tolerance.^24^ Similarly, in 2016, Sarvaria et al.^25^ reported that Bregs in cord blood, and the IL‐10 they produce, can inhibit chronic rejection of cord blood transplants. The absence of IL‐10‐secreting Bregs results in a decrease in the number of CD4^+^ Foxp3^+^ Tregs.^26^ These findings suggest that IL‐10‐secreting Bregs play a critical role in promoting immune tolerance and preventing transplant rejection.

Some scientists hold the view that Bregs facilitate immune tolerance by interacting with Tregs via transforming growth factor‐β (TGF‐β) rather than IL‐10. In 2007, Lee's research team demonstrated, using a mouse model of heart transplantation, that a group of B lymphocytes identified by the CD45RB antibody played a critical role in establishing immune tolerance. Further research by the team revealed that these B lymphocytes were Bregs, which produced TGF‐β to induce immune tolerance of Tregs. Deleting TGF‐β in Bregs resulted in the failure of immune tolerance.^27^ However, this hypothesis remains a topic of ongoing debate and has not been universally accepted by all scientists.

Further investigations are required to determine whether the interaction between Bregs and Tregs is mediated through direct contact, such as specific surface receptors, or by other cytokines.

Additionally, excessive immunosuppressive therapy following lung transplantation can result in immunodeficiency and increased susceptibility to infection. B lymphocytes may serve as a useful indicator for monitoring infection in these patients. In a study by San et al.,^28^ the number of naive B cells BM2ʹ (defined as IgD^+^ CD38^++^) located in the germinal center was positively correlated with the incidence of infection within 1 year posttransplantation in lung recipients. The increase in BM2ʹ was accompanied by a decrease in CD27− IgD− B cells. These findings may have important clinical implications for the management of posttransplantation immunosuppression and infection prevention.

### 4.2 T lymphocytes

T lymphocytes have traditionally been considered as the central cells in transplant rejection. However, in recent years, there has been growing interest among immunological researchers in the various T lymphocyte subsets. These include CD8^+^ T cells, circulating and tissue‐resident Tregs, as well as Th1, Th2, and Th17 subpopulations of CD4^+^ T cells. The investigation of these T lymphocyte subsets has led to a more detailed understanding of their roles in graft rejection.

#### 4.2.1 CD8^+^ T cells

CD8^+^ T cells, also known as cytotoxic T cells (Tc or CTL), have long been identified as the primary immune cells associated with rejection after lung transplantation. These cells are capable of directly killing target cells. Numerous studies have demonstrated a negative correlation between the number of CTL and various measures of graft function, including forced expiratory volume, total lung capacity, 50% of maximal expiratory flow after expiratory flow, oxygenation index, and bronchiolitis obliterans (BO) in lung transplant patients.^29^, ^30^, ^31^ These findings suggest that targeting CTL may be a promising therapeutic strategy for improving the outcomes of lung transplantation.

#### 4.2.2 CD4^+^ T cells

Once CD4^+^ T cells receive antigenic stimulation, they can differentiate into various subsets of T cells and perform different functions depending on the local environment. The direction of Th cell differentiation is regulated by a variety of factors, including cytokines, hormones, and other factors. The type and balance of cytokines in the local environment play a crucial role in their differentiation. CD4^+^ T cells initially induced by IL‐1, IL‐2, and IFN‐γ can differentiate into Th1 cells that secrete IFN‐γ and participate in cellular immunity. On the other hand, under IL‐4 induction, CD4^+^ T cells can differentiate into Th2 cells, which secrete IL‐4, IL‐5, and IL‐13 and are involved in humoral immunity. The presence of TGF‐β can induce CD4^+^ T cells to differentiate into Treg cells that secrete TGF‐β and participate in immune regulation. When induced by TGF‐β in conjunction with IL‐6, CD4^+^ T cells can differentiate into Th17 cells that secrete IL‐6 and IL‐17 and are involved in inflammatory responses and autoimmune diseases. These findings highlight the important role of cytokines in directing the differentiation and function of CD4^+^ T cells, and suggest the potential for targeting specific cytokines as a therapeutic strategy for immune‐related diseases.

In 1995, Sakaguchi made a groundbreaking discovery that 10% of peripheral CD4^+^ T cells in adult mice expressed the IL‐2 receptor alpha chain CD25, and were subsequently named CD4^+^ CD25^+^ regulatory T cells (Tregs). These cells were found to play a crucial role in preventing autoimmune disease in mice.

Recently, J. Salman et al. from Germany conducted a prospective study to investigate the relationship between the frequency of peripheral blood Tregs and the 2‐year incidence of CLAD in patients who underwent lung transplantation. The study measured Tregs at various time points before and after transplantation and analyzed the expression levels of CTLA4, CD127, FoxP3, and IL‐2. The results showed that a high frequency of Tregs early after transplantation was significantly positively correlated with a higher CLAD‐free rate.^32^ The study was reviewed and endorsed by Dr. Maxim Durand et al. in 2018, who suggested that the early frequency of Tregs could be used as a predictor of chronic rejection.^33^ Other studies have also shown that Tregs are closely associated with acute and chronic rejection, with most studies suggesting that the number of Tregs in the early stage after lung transplantation is positively correlated with long‐term prognosis.^2^, ^34^, ^35^, ^36^, ^37^ This suggests a potential new strategy for tolerance therapy, but also highlights the narrow therapeutic window of this treatment.

In summary, Tregs are a crucial component of the immune system that play a vital role in preventing autoimmune disease and rejection after lung transplantation. The frequency of Tregs in the early stages after transplantation may serve as a predictor of long‐term prognosis, providing a potential new avenue for tolerance therapy.

#### 4.2.3 Th17 cells

Th17 cells have garnered significant attention among researchers due to their regulatory functions. These cells are a subset of T cells that are differentiated from primordial T cells through the stimulation of TGF‐β. However, Th17 differentiation also necessitates the presence of IL‐6.^38^ In contrast to Th1 and Th2 cells, Th17 cells are adept at eliminating pathogens that are not effectively targeted by these other cell types. Th17 cells play a crucial role in the development of autoimmune diseases, as they are implicated in the pathogenesis of these conditions. Therefore, understanding the functions and regulatory mechanisms of Th17 cells is of utmost importance for researchers investigating autoimmune diseases.

Recent research has highlighted the involvement of Th17 (or IL‐17) and IL‐6 in organ transplant rejection. Of particular interest is the strong correlation between Th17 and lung transplantation. Studies have shown that the expression level of IL‐17 in BAL positively correlates with OB in human lung transplant recipients.^39^ In a study conducted by Jana and colleagues,^40^ it was discovered that patients with the A/A genotype of the IL‐17R gene rs882643 had higher PGD scores at 0, 12, and 48 h after lung transplantation. Additionally, patients with a G/G genotype in the rs2241049 region had higher PGD scores 48 h after transplantation. These findings suggest that the two loci (IL‐17R rs882643 and rs2241049) are associated with acute PGD after lung transplantation, but not with CLAD. Meanwhile, another researcher has discovered a potential link between the rs879574A genetic variant in the IL‐17A receptor gene and the development of chronic rejection and airway neutrophilia in lung transplant patients.^41^ This finding suggests that this genetic mutation may have a functional impact on the IL‐17A receptor gene, ultimately leading to the onset of CLAD following transplantation.

To further investigate the relationship between Th17 and chronic rejection in lung transplantation, several studies have used BO mice as an animal model. BO is characterized by fibroblast proliferation and cell damage caused by inflammatory infiltration, which is believed to be caused by immune factors (such as HLA mismatch) or nonimmune factors (such as infection, ischemia‐reperfusion injury, etc.). Studies have shown that IL‐6 and IL‐17 are secreted in the BO model of tracheal ectopic transplantation in mice.^42^ Specifically, IL‐6 binds to the IL‐6 receptor on the surface of naive T cells, and through the phosphorylation of JAK, STAT3, and SOCS3 signaling proteins, promotes the differentiation of T cells into Th17.^43^

Taken together, these findings suggest that IL‐17 is closely related to the occurrence of rejection after lung transplantation, both in acute rejection characterized by PGD and chronic rejection characterized by CLAD. These studies may pave the way for developing new therapeutic strategies aimed at targeting Th17 cells in lung transplant recipients.

When DCs are stimulated solely by allogeneic antigens, Th17 cells tend to secrete predominantly IL‐17A, but not IL‐17F. This IL‐17A cytokine prompts Th cells to release inflammatory cytokines, resulting in cellular damage or death. In cases where lung transplantation causes allogeneic pathogens such as CMV to invade and stimulate DCs, Th17 cells primarily secrete IL‐17F. This, in turn, activates endothelial cells and fibroblasts, promotes neutrophil proliferation and migration, and can directly contribute to BO. These findings have been documented in numerous studies and underscore the complex role of cytokines in immune responses following lung transplantation.^43^, ^44^, ^45^, ^46^, ^47^

However, Q. Wu and colleagues hold a different perspective on the production of IL‐17.^48^ According to their research, neither Th17 nor γδ T cells alone are sufficient to cause airway fibrosis. Instead, each subset may compensate for the lack of the other to produce IL‐17 and induce airway fibrosis. However, depletion of CD4^+^ T cells can reduce the production of IL‐17 and prevent OB. These findings suggest three new treatment strategies for OB. First, blocking the differentiation of naive T cells into CD4^+^ T cells or further into Th17 cells. This can be achieved by inhibiting the TGF‐β or IL‐6 signaling pathways, such as by blocking the MyD88 pathway.^49^ Second, blocking the function of IL‐17. Some substances have been found to block the production of IL‐17, including halofuginone^50^ and azithromycin,^51^ a conventional drug used to treat BO. In contrast, certain substances can promote the production of IL‐17, such as polymers of mannose on the surface of fungal cells.^52^ Last, blocking the interaction between Th17 and Th1 cells. In 2016, Yu and colleagues reported in Transplantation that blocking naive T cells with the key Th1 transcription factor T‐bet and the key Th17 transcription factor RORγT can attenuate graft‐versus‐host disease in MHC‐incompatible hematopoietic stem cell transplantation.^53^ Anchen Zhang and colleagues reported in the Journal of Heart and Lung Transplantation that silencing microRNA‐155 could effectively attenuate the interaction between Th17 cells and Th1 cells, thereby reducing chronic rejection.^54^ This study sheds light on the potential of microRNAs as therapeutic targets for preventing chronic rejection following lung transplantation.

Recent research has revealed that, in addition to CTL and Th cells, some T cells possess a memory function. Specifically, a subset of T cells known as effector memory T cells (CD62L‐) have been identified as potential candidates for T cell transfer in transplantation due to their ability to avoid inducing GVHD. In fact, CD62L‐ T cells have been shown to deplete host‐radioresistant T cells and contribute to both phenotypic and functional T‐cell reconstitution after transplantation.^55^ These findings suggest the possibility of utilizing memory T cells as a means of improving T‐cell reconstitution following transplantation, potentially leading to improved patient outcomes.

Aside from circulating memory T cells, a significant proportion of memory T cells are located within peripheral tissues and are commonly referred to as tissue‐resident memory T cells (TRM cells). As such, it is essential to consider the distinct properties of TRM cells in the context of lung transplantation. In both mouse and human studies, TRM cells have been shown to originate from heterophilic T cells that infiltrate transplanted organs. This observation highlights the potential for TRM cells to play a crucial role in preventing local infections mediated by endogenous TRM cells while also inhibiting GVHD mediated by donor TRM cells.^56^, ^57^ These findings suggest that a more comprehensive understanding of the behavior and function of TRM cells may prove instrumental in optimizing the outcomes of lung transplantation procedures.

## 5 NEUTROPHILS

Neutrophils play an active role in graft rejection and the inflammatory response, exerting a direct impact on long‐term graft function. Notably, levels of serum granulocyte colony‐stimulating factor have been shown to rise sharply within 18 h of lung transplantation but decline rapidly within 18 h of reperfusion, indicating that neutrophils may serve as a sensitive indicator of early lung function and ischemia‐reperfusion injury detection.^58^

Furthermore, neutrophil accumulation in the lungs has been observed in both mouse and human PGD. A 2014 study revealed that neutrophils release nucleic acid into the extracellular space to create a specialized structure known as neutrophil extracellular traps (NETs) in a platelet‐dependent manner, contributing to lung injury during transfusion‐related acute lung injury (TRALI). Formation of NETs is dependent on DNA released by neutrophils. In mouse and human PGD, treatment with aspirin, which can inhibit platelet or DNase I degradation of NETs, significantly reduces lung injury, suggesting that NETs represent a potential therapeutic target for PGD.^59^

## 6 OTHER IMMUNE CELLS

Aside from T cells, B cells, and neutrophils, other immune cells such as mast cells and mononuclear macrophage systems (MMS) also play a critical role in lung transplant rejection or immune tolerance.

Mast cells have been found to contribute to the accumulation of T cells, B cells, and macrophages around small blood vessels during acute rejection and ischemia‐reperfusion. They also increase tryptophan catabolism to inhibit CTL function and induce immune tolerance.^60^

MMS are critical cellular components of the immune system that participate in a diverse range of immune responses. MMS play a pivotal role in the development and progression of lung transplant rejection. The primary function of MMS in lung transplant rejection is to regulate graft antigen recognition and immune response. During the early stages of graft rejection, MMS activate immune cells by phagocytosing and presenting graft antigens, leading to the development of rejection. In the later stages of rejection, MMS participate in graft damage and repair, contributing to the progression of the rejection reaction. Therefore, the suppression of MMS activity and numbers represents a promising strategy in the management of lung graft rejection. In contrast, the role of MMS in immune tolerance is distinct from its role in rejection reactions. Specifically, MMS promote immune tolerance by delivering immune antigens and regulating the activity of CD4^+^ T cells. Moreover, MMS induce the production and activation of immunomodulatory T cells and suppress autoimmune responses, thereby promoting graft tolerance.

Recent studies have shown that intravascular nonclassical monocytes (NCMs) derived from the donor are present in the lungs of humans and mice, and they are found at the site of endothelial damage after reperfusion. Depletion of donor NCMs reduces neutrophil influx and attenuates lung graft damage. Donor NCMs produce CXCL2 through the MyD88 signaling pathway, which promotes neutrophil influx and leads to GVHD. Therefore, inhibiting the activation and chemotaxis of the MMS may be a potential strategy to alleviate GVHD.^61^, ^62^

In summary, the study of MMS in lung transplantation represents an important area of research with significant implications for the treatment of transplant rejection and the promotion of immune tolerance. By further investigating the molecular mechanisms of MMS and developing targeted interventions, we may be able to improve outcomes for patients undergoing lung transplantation.

## 7 PERSPECTIVES

The use of immunosuppressive drugs in clinical practice has significantly reduced the morbidity of hyperacute and acute rejection in lung transplant recipients. However, the long‐term survival of these patients is often impacted by chronic rejection, which is primarily manifested as BO and affects approximately 50% of recipients within the first 3 years posttransplantation.^63^ While immunosuppressants can effectively treat rejection, they may also lead to immunodeficiency and increase the risk of infection. Thus, achieving a balance between immune defense and immune tolerance is crucial for long‐term survival.

Immune cells play a vital role in regulating the immune system, and numerous studies have demonstrated that they can not only contribute to immune rejection but also induce immune tolerance. Furthermore, these cells are not only involved in attacking allografts but also in defending against invasive pathogens, highlighting their dual role in transplantation and infection immunity. Recent research has deepened our understanding of immune cell populations' functions in transplant immunity and shed light on the interplay between these cells. These findings offer insights into how immune cells can be harnessed to promote tolerance and improve long‐term outcomes for lung transplant recipients.

To date, the study of transplantation immunity has mainly focused on lymphocytes and APCs in lung transplantation. However, our understanding of immune cell networks in chronic rejection, which is characterized by BO and can lead to irreversible lung failure within 5–10 years after lung transplantation, is still incomplete. The precise role of donor or recipient immune cells in this long‐term process is yet to be fully explored. Therefore, it is critical to establish a long‐term follow‐up system after human lung transplantation and a stable animal model of chronic rejection to address this significant clinical problem. Effective long‐term follow‐up and appropriate preservation of experimental samples are also essential to support research aimed at inducing immune tolerance and improving long‐term survival in lung transplant patients. These efforts will pave the way for deeper insights into the mechanisms underlying chronic rejection and the development of novel therapeutic strategies to improve outcomes for lung transplant recipients.

## AUTHOR CONTRIBUTIONS

**Mingfeng Liao**: Formal analysis; writing—original draft. **Chaoxi Wang**: Data curation; resources. **Mingxia Zhang**: Methodology; software; writing—review and editing. **Kun Qiao**: Conceptualization; project administration; writing—review and editing.
